# *NME1* and *DCC* variants are associated with susceptibility and tumor characteristics in Mexican patients with colorectal cancer

**DOI:** 10.1186/s43046-024-00213-7

**Published:** 2024-04-01

**Authors:** Rosa María Márquez-González, Anilú Margarita Saucedo-Sariñana, César de Jesús Tovar-Jacome, Patricio Barros-Núñez, Martha Patricia Gallegos-Arreola, Mario Humberto Orozco-Gutiérrez, Ignacio Mariscal-Ramírez, Tomas Daniel Pineda-Razo, Aldo Antonio Alcaraz-Wong, María Eugenia Marín-Contreras, Mónica Alejandra Rosales-Reynoso

**Affiliations:** 1 División de Medicina Molecular, Centro de Investigación Biomédica de Occidente, Instituto Mexicano del Seguro Social (IMSS), Colonia Independencia Guadalajara, Sierra Mojada # 800, Jalisco, CP 44340 México; 2 Doctorado en Genética Humana, Centro Universitario de Ciencias de La Salud. Universidad de Guadalajara, Jalisco, México; 3 División de Genética, Centro de Investigación Biomédica de Occidente, Instituto Mexicano del Seguro Social (IMSS), Guadalajara, Jalisco México; 4 Servicio de Oncología Médica, Hospital de Especialidades, Instituto Mexicano del Seguro Social (IMSS), Guadalajara, Jalisco México; 5 Servicio de Anatomía Patológica, Hospital de Especialidades, Instituto Mexicano del Seguro Social (IMSS), Guadalajara, Jalisco México; 6 Servicio de Gastroenterología, Hospital de Especialidades, Instituto Mexicano del Seguro Social (IMSS), Guadalajara, Jalisco México

**Keywords:** Colorectal cancer, *NME1* genetic variants, *DCC* genetic variants, Susceptibility, TNM stage, Tumor location

## Abstract

**Background:**

Colorectal cancer (CRC) ranks third in cancer incidence globally and is the second leading cause of cancer-related mortality. The nucleoside diphosphate kinase 1 *(NME1)* and netrin 1 receptor *(DCC)* genes have been associated with resistance against tumorigenesis and tumor metastasis. This study investigates the potential association between *NME1* (rs34214448 G > T and rs2302254 C > T) and *DCC* (rs2229080 G > C and rs714 A > G) variants and susceptibility to colorectal cancer development.

**Methods:**

Samples from 232 colorectal cancer patients and 232 healthy blood donors underwent analysis. Variants were identified using polymerase chain reaction-restriction fragment length polymorphism (PCR–RFLP) methodology. Associations were assessed using odds ratios (OR), and the *p* values were adjusted with Bonferroni test.

**Results:**

Individuals carrying the G/T and T/T genotypes for the *NME1* rs34214448 variant exhibited a higher susceptibility for develop colorectal cancer (OR = 2.68, 95% CI: 1.76–4.09, *P* = 0.001 and OR = 2.47, 95% CI: 1.37–4.47, *P* = 0.001, respectively). These genotypes showed significant associations in patients over 50 years (OR = 2.87, 95% CI: 1.81–4.54, *P* = 0.001 and OR = 2.99, 95% CI: 1.54–5.79, *P* = 0.001 respectively) and with early Tumor-Nodule-Metastasis (TNM) stage (*P* = 0.001), and tumor location in the rectum (*P* = 0.001). Furthermore, the *DCC* rs2229080 variant revealed that carriers of the G/C genotype had an increased risk for develop colorectal cancer (OR = 2.00, 95% CI: 1.28–3.11, *P* = 0.002) and were associated with age over 50 years, sex, and advanced TNM stages (*P* = 0.001).

**Conclusions:**

These findings suggest that the *NME1* rs34214448 and *DCC* rs2229080 variants play a significant role in colorectal cancer development.

## Introduction

Colorectal cancer (CRC) is ranked as the third most diagnosed cancer globally and contributes significantly to cancer-related mortality worldwide [1]. According to Globocan 2022, the incidence of CRC in Mexico in 2022 showed a ratio of 12.2 per 100,000 individuals, with a mortality ratio of 6.3 per 100,000 inhabitants [1]. The development of CRC involves a multifactorial combination of events, including genetic alteration and epigenetic changes, leading to abnormalities in the normal colonic mucosa. Inactivation of tumor suppressor genes through deletion or mutation in the remaining allele plays a significant role in cancer progression and metastasis [2]. The process of metastasis itself is complex and includes several stages, the molecular regulation of which remains poorly understood. Tumor suppressor genes as nucleoside diphosphate kinase 1 *(NME1)* and netrin 1 receptor *(DCC)* are crucial in the development of metastasis [3, 4].

The *NME1* gene, located on chromosome 17q21, consists of 6 exons and encodes the NME1 protein, also known as NME1-H1 or NM23-H1, which is member of a family of 10 *NME1* genes [5]. Numerous research studies have demonstrated that *NME1* functions as a metastasis suppressor gene, exhibiting suppressive activity across a wide range of human cancers. NME1 is involved in various processes, including transcription, regulation of deoxyribonucleic acid (DNA) replication, and cell differentiation, both in normal and tumorigenic cells [6, 7]. Additionally, NME1, along with TP53 and MDM2, plays a significant role in the ERK and K-RAS signaling pathways. Changes in *NME1* expression levels result in decreased activation of the ERK signaling pathway [8].

The *DCC* gene, composed of 29 exons, is located on chromosome 18q21. It encodes the Netrin 1 receptor, a transmembrane receptor belonging to the immunoglobulin superfamily. Neurons abundantly express DCC and plays roles in promoting cell survival and axon regeneration [9]. Research indicates that this gene functions as both a tumor suppressor and a metastasis suppressor. It is involved in various cellular processes including cell division, migration, apoptosis, cell cycle arrest, cell differentiation, embryonic development, and metastasis. Studies suggest that loss of heterozygosity (LOH) and other alterations in DCC are linked with poor differentiation, metastasis, and an unfavorable prognosis in CRC [10–13].

The impact of *NME1* rs34214448 (c.-5 + 989G > T) and rs2302254 (c.-128C > T) variants, as well as *DCC* rs2229080 (c.-601G > C) and rs714 A > G (intronic) variants on cancer susceptibility, prognosis, or survival has been investigated across various cancers types, including esophageal, gastric adenocarcinoma, gallbladder, breast, cervicouterine, ovarian, leukemia, and colorectal cancer [14–22]. However, only two studies have been conducted on the *NME1* rs34214448 variant in the Caucasian population [18, 23] and the *DCC* rs2229080 variant in the Indian population for colorectal cancer [24].

This study aims to investigate, for the first time, the allele and genotype distribution of the *NME1* gene (rs2302254) and the *DCC* gene (rs2229080 and rs714) variants; and to assess their potential association of *NME1* (rs34214448 and rs2302254) and the *DCC* (rs2229080 and rs714) of this gene variants with the clinicopathological characteristics and the development of CRC in Mexican patients.

## Materials and methods

### Study population

This study protocol underwent evaluation by the Scientific Research Committee of the Mexican Social Security Institute (IMSS) and was approved with the register number: R-2018–1305-001. All procedures were conducted in accordance with national and international ethical standards. Informed consent forms were obtained from each participant, both patients and controls, before the commencement of the study, authorizing their participation and the collection of blood samples. A total of 464 individuals were included in the study: 232 patients diagnosed with sporadic colorectal adenocarcinoma, confirmed through clinical features and histological examination, according with the Clinical Practice Guidelines on Colon and Rectal Cancer, as well as the clinicopathologic criteria from the Hospital de Especialidades del IMSS in Guadalajara, Mexico. Patients were enrolled in a non-probabilistic consecutive manner during consultation at this institution between 2018 to 2022. Clinical stages of CRC were based on Tumor-Nodule-Metastasis (TNM) classification system. Blood samples were collected during clinical visits before the initiation of chemotherapy or radiotherapy. The control group comprised 232 unrelated subjects (109 men and 123 women), with negative findings for malignancy upon colonoscopy. For both groups the age was matched. Exclusion criteria for both patients and controls included a diagnosis of autoimmune or inflammatory bowel disease (Crohn’s disease and ulcerative colitis), as well as a known history of hereditary cancer. The entire study population resided in the metropolitan area of Guadalajara, Mexico, and all belonged to the mixed-race category. Clinical information, including age, sex, smoking and drinking habits, family history, symptoms, and clinicopathological characteristics, was obtained by consulting hospital medical records.

### Genotyping

Genomic DNA from peripheral blood lymphocytes was isolated using the Miller, Dykes & Polesky, method [25]. The rs34214448 (G > T) and rs2302254 (C > T) variants of *NME1* gene and rs2229080 (G > C) and rs714 (A > G) of *DCC* gene, were genotyped by polymerase chain reaction–restriction fragment length polymorphism (PCR–RFLP) method. For *NME1* (rs34214448) variant was performed using the forward primer F: 5´- CCC ACC GTT TAT TGG CTA G-3´ and the reverse primer 5´- CAA CCC CCT TCA TTT TAC AA-3´ [17] and for *NME1* rs2302254 variant with the forward primer 5´- CGC GAA CGA AGG AAG TGA GTC A-3´ and reverse primer 5´- GCC GCC AGC ACC CGA AAC-3´ [25]. For *DCC* (rs2229080) variant was performed using the forward primer F: 5´- ATT TGG AAG ACT TAT TCT TCC-3´ and the reverse primer 5´- CGG TAA ATT CCA AGT CCC TCG GTT GGA GC-3´ and for *DCC* rs714 variant with the forward primer 5´- TGC ACC ATG CTG AAG ATT GT-3´ and reverse primer 5´- AGT ACA ACA CAA GGT ATG TG-3´ [21]. PCR reaction for *NME1* (rs34214448 and rs2302254) and DCC (rs2229080 and rs714) variants was performed for 35 cycles in a 10 μL volume containing 100 ng DNA, 10X buffer (500 mM KCl, 100 mM Tris–HCl, and 0.1% Triton™ X-100), 2.0 mM MgCl2, 200 mM dNTPs, 1 pM of each primer, and 2U Taq DNA Polymerase (Thermo Fisher Scientific, USA) according to the following amplification program: initial denaturation at 94 °C for 5 min, 32 cycles of denaturation at 94 °C for 30 s, annealing at 58℃ for (rs34214448), 62℃ for (rs2302254), 58℃ for (rs2229080) and 56℃ for (rs714) respectively for 30 s, and extension at 72 °C for 30 s followed by final extension at 72 °C for 10 min. Five microliters of each PCR products were digested using 5U of the next restriction enzymes: *NME1* rs34214448 (*EcoRI*) and rs2302254 (*BanII*) and for *DCC* rs2229080 and rs714 (*MspI*) (New England Biolabs, USA) according to the manufacturer’s instructions and finally separated in a 6% polyacrylamide gels. Genotypes was identified according to described by [17, 21, 26]. To ensure the quality of the genotyping processes, approximately 10% of the randomly samples were reprocessed, and the results were found to be 100% consistent.

### Statistical analysis

The T-student test was used to compare the means of age between patients and controls. Non-parametric Chi-square test was used to calculate the Hardy–Weinberg Equilibrium (HWE) of each variant and for demographic variables (age, sex, alcohol, and tobacco status) between the groups. Genotypic and allelic frequencies were calculated by direct counting for each group. Odds ratio (OR) and respective 95% confidence intervals (CI) analyses were used to associate genotypic and allelic frequencies with demographic and clinicopathological characteristics. A binary logistic regression analysis was performed to evaluate the confounding variables. The T-student, Chi-square, and Logistic regression tests were conducted using SPSS v.28.0 software (SPSS Inc., Chicago, IL, USA), while odds ratios and CIs were calculated using Epi Info. A significance level of *P* < 0.05 was considered statistically significant for the analyzed parameters. Additionally, the Bonferroni test was employed as an adjustment method to correct values (*P* < 0.0125).

## Results

### Demographic and clinical features of the study groups

The demographic, clinical, and anatomopathological information of CRC patients and healthy control subjects enrolled is presented in Table 1. The CRC group comprised 232 patients (134 male and 98 female), while the control group consisted of 232 healthy subjects (109 male and 123 female). All participants were efficiently genotyped for *NME1* (rs34214448 G > T and rs2302254 C > T) and *DCC* (rs2229080 G > C and rs714 A > G) variants. The mean ages were 60.73 years (range 40 to 92) and 62.45 years (range 43 to 92) for the CRC and control group, respectively. Significant differences (*P* = 0.001) were observed in the smoking and drinking status between the two groups. Regarding clinical and pathological characteristics, 70.2% had stage III-IV tumors, 32.7% had metastases, and 53.45% had tumors located in the rectum.

**Table 1:** Clinicopathological features of the colorectal cancer patients and control subjects

Characteristic	CRC group	Control group	*P value*
	***n* = 232 (100%)**	***n* = 232 (100%)**	
**Mean Age (years SD)**	60.73 (± 10.25)	62.45(± 12.39)	0.103
**Age (in years)**			
< 50	28 (12.07)	44 (18.97)	0.040
> 50	204 (87.93)	188 (81.03)	
**Sex**			
Male	134 (57.76)	109 (46.98)	0.020
Female	98 (42.24)	123 (53.02)	
**Smoking status**			
Yes	79 (34.05)	37 (15.95)	**0.001**
No	153 (65.95)	195 (84.05)	
**Drinking status**			
Yes	65 (28.02)	29 (12.50)	**0.001**
No	167 (71.98)	203 (87.50)	
**Clinical stage TNM**			
I	6 (2.59)		
II	63 (27.16)		
III	87 (37.5)		
IV	76 (32.76)		
**Tumor location**			
Colon	108 (46.55)		
Rectum	124 (53.45)		
**Site of metastasis**			
Liver	29 (38.16)		
Lung	13 (17.11)		
Liver and lung	8 (10.53)		
Peritoneum	3 (3.95)		
Lung and peritoneum	3 (3.95)		
Ovary	1 (1.32)		
Brain	1 (1.32)		
No Available	18 (23.68)		
**Treatment response**			
Complete response	101 (43.53)		
Partial response	68 (29.31)		
No response	63 (27.16)		

*P*-value were adjusted by the Bonferroni test (0.0125); Bold text highlights statistically significant findings

### *NME1 *and* DCC* variants in CRC patients and control subjects

In the control group, all four analyzed SNPs was found to be in Hardy–Weinberg Equilibrium (HWE) (*p* > 0.05). HWE for the *NME1* rs34214448 and rs2302254 variants were X2 = 0.043 (*P* = 0.83) and X2 = 1.808 (*P* = 0.17), respectively. While the HWE for the *DCC* rs2229080 and rs714 variants were X2 = 1.887 (*P* = 0.16) and X2 = 1.048 (*P* = 0.30), respectively. Frequencies of the *NME1* rs34214448 and *DCC* rs2229080 variants exhibited significant differences between CRC patients and control individuals (Table 2). The *NME1* rs34214448 genotype G/T was observed in 62.07% (144/232) of the CRC patients and 44.83% (104/232) of the controls, indicating a statistically significant difference (OR = 2.68; 95% CI = 1.76–4.09, *P* = 0.001). Likewise, the genotype T/T was found in 15.9% (37/232) of the CRC patients and 12.5% (29/232) of the controls, indicating a statistically significant difference (OR = 2.47; 95% CI = 1.37–4.47, *P* = 0.001). When considering the dominant allelic interaction pattern of G/T + T/T versus G/G, the T allele demonstrated a statistically significant correlation with CRC (OR = 2.06; 95% CI = 1.38–3.07, *P* = 0.001). Furthermore, there were significant differences in allele frequencies between the groups; individuals carrying the T allele showed an elevated predisposition to CRC (OR = 1.65; 95% CI = 1.26–2.15, *P* = 0.001). Concerning to *NME1* rs2302254 C > T variant no statistical significance was observed between the groups.

**Table 2:** Distribution of genotypes and allelic frequencies of the *NME1* rs34214448 and rs2302254 and *DCC* rs2229080 and rs714 variants in CRC and control group

Genotype	CRC group *n* = 232 (%)	Control group *n* = 232 (%)	OR (95% CI)	*P value*
***NME1* (rs34214448) G > T**				
**G/G**	51 (21.98)	99 (42.67)	1.00 (Reference)	
**G/T**	144 (62.07)	104 (44.83)	**2.68 (1.76- 4.09)**	**0.001**
**T/T**	37 (15.95)	29 (12.50)	**2.47 (1.37- 4.47)**	**0.001**
**G/T + T/T vs. G/G**	181 (78.02)	170 (57.33)	**2.06 (1.38 – 3.07)**	**0.001**
**Allele**				
**G**	246 (53.02)	302(65.09)	1.00 (Reference)	
**T**	218 (46.98)	162(34.91)	**1.65 (1.26 – 2.151)**	**0.001**
***NME1* (rs2302254) C > T**				
**C/C**	54 (23.28)	65 (28.02)	1.00 (Reference)	
**C/T**	124 (53.45)	125 (53.88)	1.19 (0.77 – 1.85)	0.495
**T/T**	54 (23.28)	42 (18.10)	1.54 (0.90 – 2.65)	0.147
**C/T + T/T vs. C/C**	178 (76.72)	167 (71.98)	1.28 (0.82 – 1.99)	0.287
**Allele**				
**C**	232 (50.0)	255 (54.96)	1.00 (Reference)	
**T**	232 (50.0)	209 (45.04)	1.22 (0.94 – 1.57)	0.148
***DCC* (rs2229080) G < C**				
**G/G**	48 (20.69)	78 (33.62)	1.00 (Reference)	
**G/C**	128 (55.17)	104 (44.83)	**2.00 (1.28 – 3.11)**	**0.002**
**C/C**	56 (24.14)	50 (21.55)	1.82 (1.07 – 3.07)	0.034
**G/C + C/C vs. G/G**	184 (79.31)	154 (66.38)	**1.94 (1.27 – 3.02)**	**0.002**
**Allele**				
**G**	224 (48.28)	260 (56.03)	1.00 (Reference)	
**C**	240 (51.72)	204 (43.97)	1.36 (1.05 – 1.76)	0.021
***DCC* (rs714) A > G**				
**A/**A	45 (19.40)	57 (24.57)	1.00 (Reference)	
**A/G**	130 (56.03)	108 (46.55)	1.52 (0.95—2.43)	0.097
**G/G**	57 (24.57)	67 (28.88)	1.07 (0.63—1.82)	0.885
**A/G + G/G vs. A/A**	187 (80.60)	175 (75.43)	1.35 (0.87 – 2.10)	0.217
**Allele**				
**A**	220 (47.41)	222 (47.41)	1.00 (Reference)	
**G**	244 (52.59)	242 (52.59)	1.01 (0.78—1.31)	0.947

*P*-value were adjusted by the Bonferroni test (0.0125); Bold text highlights statistically significant findings

For the *DCC* rs2229080 G > C variant, significant differences were observed between patients versus control individuals for the G/C genotype (OR = 2.00; 95% CI = 1.28–3.11, *p* = 0.002). When considering a dominant pattern of allelic interaction (G/C + C/C vs. G/G), the C allele showed an association with CRC (OR = 1.94; 95% CI = 1.27–3.2, *p* = 0.002). Regarding to rs714 G > C variant in the *DCC* gene, no statistical significance was observed between the groups.

### *NME1* and *DCC* genotypes analysis by clinicopathological characteristics

The association between genotypes *NME1* rs34214448 and rs2302254 and *DCC* rs2229080 and rs714 with clinicopathological characteristics are shown in Tables 3, 4, 5 and 6. Individuals over the age of 50 carrying the T/T and G/T genotypes of *NME1* rs34214448 variant are more susceptible to developing CRC (OR = 2.99, 95% CI = 1.54–5.79, *P* = 0.001, and OR = 2.87, 95% CI = 1.81–4.54, *P* = 0.001 respectively). Women with the G/T genotype have a significantly higher risk of developing CRC (OR = 3.05, 95% CI = 1.59–5.81, *P* = 0.001). Furthermore, the dominant GT + TT vs G/G model indicates an increased susceptibility in women to develop CRC (OR = 2.94, 95% CI = 1.57–5.50, *P* = 0.001).

**Table 3:** Association of the *NME1* rs34214448 variant with clinicopathological characteristics

*NME1* rs34214448						
**CRC/Control**				**OR (95% CI); *P* value**		
**Variable**	**GG**	**GT**	**TT**	**TT versus GG**	**GT versus GG**	**GT + TT versus GG**
**Age (years)**						
< 50	8/17	16/19	4/8	1.06 (0.24–4.59); 1.000	1.78 (0.61–5.22); 0.422	1.57 (0.56–4.36); 0.534
> 50	43/82	128/85	33/21	**2.99 (1.54–5.79); 0.001**	**2.87 (1.81–4.54); 0.001**	**2.89 (1.85–4.51); 0.001**
**Sex**						
Male	33/50	79/46	22/13	2.56 (1.13–5.78); 0.036	**2.60 (1.47–4.60); 0.001**	**2.59 (1.50–4.47); 0.001**
Female	18/49	65/58	15/16	2.55 (1.05–6.20); 0.061	**3.05 (1.59–5.81); 0.001**	**2.94 (1.57–5.50); 0.001**
**TNM stage**						
I + II	15/99	44/104	10/29	2.27 (0.92–5.60); 0.116	**2.79 (1.46–5.33); 0.002**	**2.67 (1.42–5.02); 0.002**
III + IV	36/99	100/104	27/29	**2.56 (1.33–4.89); 0.006**	**2.64 (1.65–4.23); 0.001**	**2.62 (1.67–4.12); 0.001**
**Localization**						
Colon	28/99	70/104	10/29	1.21 (0.53–2.80); 0.803	**2.37 (1.41–3.99); 0.001**	**2.12 (1.28–3.51); 0.004**
Rectum	23/99	74/104	27/29	**4.00 (2.00–8.01); 0.001**	**3.06 (1.77–5.27); 0.001**	**3.26 (1.93–5.51); 0.001**

*P*-value were adjusted by the Bonferroni test (0.0125). Bold text highlights statistically significant findings

**Table 4:** Association of the *NME1* rs2302254 variant with clinicopathological characteristics

*NME1* rs2302254						
		**CRC/Control**			**OR (95% CI); *P* value**	
**Variable**	**CC**	**CT**	**TT**	**TT versus CC**	**CT versus CC**	**CT + TT versus CC**
**Age (years)**						
< 50	3/15	17/23	8/6	6.66 (1.30–34.02); 0.043	3.69 (0.92–14.82); 0.106	4.31 (1.11–16.62); 0.050
> 50	51/50	107/102	46/36	1.25 (0.69–2.24); 0.544	1.02 (0.63–1.65); 1.000	1.08 (0.69–1.70); 0.806
**Sex**						
Male	36/36	63/55	35/18	1.94 (0.93–4.04); 0.108	1.14 (0.63–2.05); 0.761	1.34 (0.77–2.33); 0.365
Female	18/29	61/70	98/24	**6.57 (3.14–13.76); 0.001**	1.40 (0.71–2.77); 0.419	2.72 (1.43–5.17); 0.002
**TNM stage**						
I + II	14/65	36/125	19/42	2.10 (0.95–4.63); 0.097	1.33 (0.67–2.65); 0.507	1.52 (0.79–2.93); 0.260
III + IV	40/65	88/125	35/42	1.35 (0.74–2.46); 0.398	1.14 (0.70–1.84); 0.668	1.19 (0.75–1.89); 0.512
**Localization**						
Colon	24/65	51/125	32/42	2.06 (1.07–3.97); 0.044	1.10 (0.62–1.95); 0.842	1.34 (0.78–2.30); 0.340
Rectum	29/65	73/125	22/42	1.17 (0.59–2.30); 0.770	1.30 (0.77–2.21); 0.380	1.27 (0.76–2.11); 0.413

*P*-value were adjusted by the Bonferroni test (0.0125). Bold text highlights statistically significant findings

**Table 5:** Association of the *DCC* rs2229080 variant with clinicopathological characteristics

*DCC* rs2229080 G > C						
	**CRC/Control**			**OR (95% CI); *P* value**		
**Variable**	**GG**	**GC**	**CC**	**CC versus GG**	**GC versus GG**	**GC + CC versus GG**
**Age (years)**						
< 50	13/14	9/17	6/13	0.49 (0.14–1.69); 0.412	0.57 (0.18–1.72); 0.471	0.46 (0.17–1.26); 0.131
> 50	35/64	119/87	50/37	0.84 (0.29–2.41); 0.957	**2.50 (1**.**52–4.10); 0.001**	**2.28 (1.40–3.72); 0.001**
**Sex**						
Male	26/43	75/48	33/18	**3.03 (1.42–6.43); 0.006**	**2.58 (1.40–4.74); 0.002**	**2.70 (1.52–4.81); 0.001**
Female	22/35	53/56	23/32	1.14 (0.53–2.43); 0.876	1.50 (0.78–2.89); 0.217	1.37 (0.74–2.54); 0.390
**TNM stage**						
I + II	16/78	33/104	20/50	1.95 (0.92–4.11); 0.114	1.54 (0.79–3.00); 0.259	1.67 (0.90–3.12); 0.135
III + IV	28/78	95/104	36/50	2.00 (1.09–3.68); 0.035	**2.54 (1.52–4.25); 0.001**	**2.36 (1.45–3.87); 0.001**
**Localization**						
Colon	21/78	61/104	25/50	1.76 (0.89–3.48); 0.143	2.06 (1.15–3.68); 0.019	1.96 (1.13–3.41); 0.021
Rectum	26/78	67/104	31/50	1.86 (0.98–3.49); 0.075	1.93 (1.12–3.31); 0.022	1.90 (1.14–3.18); 0.017

*P*-value were adjusted by the Bonferroni test (0.0125). Bold text highlights statistically significant findings

**Table 6:** Association of the *DCC* rs714 variant with clinicopathological characteristics

*DCC* rs714 A < G						
	**CRC/Control**			**OR (95% CI); *P* value**		
**Variable**	**AA**	**AG**	**GG**	**GG versus AA**	**AG versus AA**	**AG + GG versus AA**
**Age (years)**						
< 50	3/12	21/21	4/11	1.45 (0.26–8.00); 1.000	4.00 (0.98–16.25); 0.086	3.12 (0.79–12.28); 0.164
> 50	42/45	109/87	53/56	1.01 (0.57–1.78); 1.000	1.34 (0.80–2.22); 0.311	1.21 (0.75–1.95); 0.499
**Sex**						
Male	32/28	73/48	29/33	0.76 (0.37–1.56); 0.586	1.33 (0.71–2.48); 0.460	1.10 (0.61–1.97); 0.86
Female	13/29	57/60	28/34	1.83 (0.80–4.18); 0.211	2.11 (1.00–4.47); 0.070	2.01 (0.98–4.13); 0.077
**TNM stage**						
I + II	16/57	32/108	21/67	1.11 (0.53–2.34); 0.917	1.05 (0.53–2.08); 1.000	1.07 (0.57–2.03); 0.940
III + IV	29/57	73/108	40/67	1.17 (0.64–2.12); 0.706	1.32 (0.77–2.27); 0.366	1.26 (0.76–2.10); 0.424
**Localization**						
Colon	19/57	64/108	25/67	1.11 (0.55–2.23); 0.886	1.77 (0.97–3.25); 0.08	1.52 (0.85–2.72); 0.194
Rectum	26/57	66/108	32/67	1.04 (0.55–1.95); 1.000	1.32 (0.76–2.33); 0.371	1.22 (0.72–2.07); 0.526

*P*-value were adjusted by the Bonferroni test (0.0125). Bold text highlights statistically significant findings

The analysis of disease development reveals that women in early TNM stages (I + II) who carry the G/T genotype are more susceptible to developing CRC (OR = 2.79, 95% CI = 1.26–4.80, *P* = 0.002). Based on tumor location analysis, individuals with the T/T genotype are more susceptible to developing tumors in the rectum (OR = 4.00; 95% CI = 2.00–8.01, *P* = 0.001) (see Table 3). We found statistical significance only in female patients carrying T/T genotype of *NME1* rs2302254 variant (OR = 6.57; 95% CI = 3.14–13.76, *P* = 0.001). Regarding age, TNM stage, and tumor location no statistical association was observed (Table 4).

For the *DCC* rs2229080 variant, individuals over 50 carrying the G/C genotype demonstrated a higher susceptibility to develop CRC (OR = 2.50, 95% CI = 1.52–4.10, *P* = 0.001). This association was also observed with the dominant GC + CC vs G/G model of inherited disease (OR = 2.28, 95% CI = 1.40–3.72, *P* = 0.001). Patients in advanced TNM stages (III + IV) and carrying the G/C genotype exhibit greater susceptibility to developing CCR (OR = 2.54, 95% CI = 1.52–4.25, *P* = 0.001). No statistically significant differences were observed in the analysis of tumor location (Table 5). Furthermore, no statistical association was found for the *DCC* rs714 variant when comparing sex, age, TNM stage, and tumor location among groups (Table 6).

### Multivariable logistic regression analysis with confounding variables

The results of the multiple logistic regression analysis are shown in Table 7 including confounding variables. Statistical significance was observed for tobacco and alcohol use in the presence of the two variants associated in *NME1* (rs34214448) and *DCC* (rs2229080) (OR = 2.10; 95% CI = 1.30–3.37; *P* = 0.002 and OR = 2.06; 95% CI = 1.23–3.46; *P* = 0.006, respectively), suggesting that these variables increase the risk and susceptibility of developing CRC.

**Table 7:** Logistic Regression Analysis for the *NME1* and *DCC* variants analyzed with confounding variables

Independent Variable	Regression Coefficient	Standard Error	Wald Test	Degrees of Freedom	*P value*	OR (95% IC)
**Age** ** > 50 vs. < 50**	0.442	0.280	2.482	1	0.115	1.55 (0.89 -2.69)
**Sex** **Male vs. Female**	0.422	0.201	4.428	1	0.035	1.52 (1.02 -2.26)
**Smoking status** **Yes vs. No**	0.742	0.243	9.358	1	**0.002**	**2.10 (1.30 -3.37)**
**Drinking status** **Yes vs. No**	0.725	0.264	7.522	1	**0.006**	**2.06 (1.23 -3.46)**
*NM23* rs34214448 **GT + TT**	0.858	0.217	15.688	1	**0.001**	**2.35 (1.54 -3.60)**
*DCC* rs2229080 **GC + CC**	0.637	0.228	7.806	1	**0.005**	**1.89 (1.21–2.95)**
**Constant**	-1.862	0.307	36.873	1	**0.001**	
**Model**	X^2^ = 63.475 d.f. = 6 P = **0.001**					

Bonferroni test was used to adjust the *P* value (0.0125); Bold values indicate statistically significant findings

## Discussion

Colorectal cancer is a complex disease influenced by multiple factors such as genetics, epigenetics, and environmental factors. Variants in two genes linked to CRC, *NME1* (rs34214448 and rs2302254), and *DCC* (rs2229080 and rs7124), have been examined in various cancer types and populations, yielding inconsistent findings.

In this study conducted in the Mexican population, there is a clear increase in CRC incidence among individuals over the age of 50 (87.93%). This finding has been previously reported in other studies and aligns with global statistics [27, 28]. Significant statistical differences were observed between the groups in terms of tobacco and alcohol use. These results are consistent with those reported by Tsong et al. in 2007 and Minami et al. in 2022, which highlighted the important role that alcohol and tobacco consumption play in the development of colorectal carcinogenesis in the Chinese population, demonstrating that individuals who consume more than 7 alcoholic drinks a week present a high risk up to 72% over non-drinkers, similarly, it was observed that smokers present an increased risk of rectal cancer over non-smoking individuals [29, 30]. Yang LP et al. in 2021 found a significant increase among CRC patients who smoked, especially with left laterality, also associated with a longer duration and higher rate of smoking, with the risk being up to 55% higher in non-smokers [31]. In another study, Huang et al. in 2022, referred smoking is associated not only with the development of CRC, but also with a high risk of mortality in this entity, being 1.11 times higher than non-smoking patients. This risk was also present in patients who smoked more than 12 cigarettes per day or for more than 30 years [32]. In the study carried out by Botteri, it is also mentioned that the risk of CRC increases linearly with the intensity and duration of smoking and was also mainly associated with variants in *BRAF* and microsatellite instability rather than with variants in *KRAS* or *TP53* [33].

The *NME1* gene was the first identified metastasis suppressor gene. Low expression of *NME1* has been linked to metastasis in various tumors, including melanoma, breast cancer, prostate cancer, and colorectal cancer. However, the mechanism responsible for this association is not fully understood [14, 34]. The most extensively studied variants within the promoter region of the *NME1* gene are rs16949649 and rs2302254, which have been associated with low expression [35]. However, the rs34214448 variant, located in intron 1, has been implicated in possible modifications of post-transcriptional processes [35].

Analysis of genotypic and allelic frequencies for the *NME1* rs34214448 G > T variant revealed significant differences (*P* = 0.001). The G/T and T/T genotypes were found to be significantly associated with an increased risk of CRC; this could confirm their role as a risk factor for CRC susceptibility.

Additionally, females over 50 years old showed an association with early TNM stages (I and II), and the rectum location was detected most frequently in these patients. These findings are the first reported among Mexican patients with CRC. However, Stremitzer S, et al. (2015) reported a similar association with recurrence-free survival (RFS) in patients with resected colorectal liver metastases [18]. Furthermore, our findings align with prior research on cervical neoplasia [14], lung cancer [35], breast cancer [21], and gastric cancer [15]. No significant differences were observed for the rs2302254 variant of the *NME1* gene; however, it has been associated with other cancers [15, 36–38].

*DCC* is a transmembrane protein that belongs to the immunoglobulin superfamily. It has been identified as a potential tumor suppressor gene located on chromosome 18q21. DCC is expressed in normal tissues as colonic mucosa, however, in CRC samples analyzed the protein has been missing. DCC plays an essential role in directing cell invasion through the basement membrane, making it a crucial component in the pathological progression of human cancer [19, 39].

*DCC* deletions have been found in 20–75% of CRC, and gene alterations are linked to cancer and metastasis [39]. The most extensively studied *DCC* variants are rs714, rs4078288, and rs7504990, which are situated in an intronic region and could affect expression levels. Meanwhile, the rs2229080 variant is a missense mutation found in exon 3 that replaces Arg (CGA) to Gly (GGA), at codon 201 of DCC protein [9]. This variation has been linked to decreased expression regulation and has been clinically associated with advanced cancer stages, a poor prognosis, and increased susceptibility to CRC [19, 40]. Bakshi et al. have reported that the presence of the C allele is a protective factor in breast cancer and found that the ribonucleic acid (RNA) secondary structure of wild-type alleles enhances their stability [9].

Significant differences were observed between groups regarding the rs2229080 G > C variant. The G/C genotype was found to be significantly associated with an increased risk of developing colorectal cancer, indicating that it is a genetic factor for susceptibility to CRC. Additionally, we have identified a significant correlation between male patients over 50 years old and those with an advanced TNM stage (III + IV). These results are consistent with those reported by Sharma et al., 2021 in patients with CRC [24], as well as breast and gallbladder cancer [39]. On the other hand, no association has been observed for the rs2229080 variant with esophageal and gastric cancer [19]. However, in two studies, the C allele has been reported as a protective factor against breast cancer [9, 39].

In relation to the *DCC* rs714 variant, no significant association was detected in the present study with any demographic and clinicopathological variables. However, in a few reports a positive association has been observed. For instance, Malik et al. found an association with esophageal and gastric cancer [17], and Rai et al. reported an association with gallbladder cancer [40].

Finally, the multivariable analysis showed, for the first time, that tobacco and alcohol consumption are a risk factor for CRC in carriers of the G/T or T/T genotypes of the rs34214448 *NME1* variant, and G/C or C/C genotypes of the rs2229080 *DCC* variant. Potentially modifiable lifestyle factors, such as tobacco and alcohol consumption, have been associated with CRC risk in studies conducted in Western populations [41].

Further studies with larger sample sizes, functional genomics and analysis in other populations are needed to validate the genetic effects of *NME1* and *DCC* polymorphisms in CRC. A limitation of this study was the absence of Body Mass Index (BMI) and follow-up data in these patients. In the future, more studies concerning to the association between other SNPs in the *NME1* and *DCC* genes and the risk of CRC are required.

## Conclusions

In conclusion, this study represents the first to identify the *NME1* rs34214448 and *DCC* rs2229080 variants as potential markers for CRC risk. Additionally, these polymorphic variants were found to be associated with age, sex, TNM stages, and tumor localization.

## Data Availability

The datasets used during the present study are available from the corresponding author upon request.

## Abbreviations

CRC: Colorectal cancer
PCR–RFLP: Polymerase chain reaction-restriction fragment length polymorphism
OR: Odds ratio
DNA: Deoxyribonucleic acid
LOH: Loss of heterozygosity
IMSS: Mexican Social Security Institute
TNM: Tumor-Nodule-Metastasis
HWE: Hardy–Weinberg Equilibrium
CI: Confidence intervals
RNA: Ribonucleic acid
